# Patients' Perception of Quality of Pre-Operative Informed Consent in Athens, Greece: A Pilot Study

**DOI:** 10.1371/journal.pone.0008073

**Published:** 2009-11-26

**Authors:** Matthew E. Falagas, Patrick D. Akrivos, Vangelis G. Alexiou, Vasilios Saridakis, Theofanis Moutos, George Peppas, Barbara K. Kondilis

**Affiliations:** 1 Alfa Institute of Biomedical Sciences (AIBS), Athens, Greece; 2 Department of Medicine, Tufts University School of Medicine, Boston, Massachusetts, United States of America; 3 Hellenic American University, Athens, Greece; 4 Department of Surgery, Agia Barbara General Hospital of West Attica, Athens, Greece; 5 Department of Ophthalmology, Henry Dunant Hospital, Athens, Greece; 6 Department of Surgery, Henry Dunant Hospital, Athens, Greece; Dalhousie University, Canada

## Abstract

**Background:**

We sought to perform a study to record and evaluate patients' views of the way surgeons communicate informed consent (IC) in Greece.

**Methodology/Principal Findings:**

A prospective pilot study was carried out in Athens from 9/2007 to 4/2008. The study sample was extracted from patients, operated by eight different surgeons, who volunteered to fill in a post-surgery self-report questionnaire on IC. A composite delivered information index and a patient-physician relationship index were constructed for the purposes of the analysis. In total, 77 patients (42 males) volunteered to respond to the questionnaire. The delivered information index scores ranged from 3 to 10, the mean score was 8, and the standard deviation (SD) was 1.9. All patients were aware of their underlying diagnosis and reason for surgery. However, a considerable proportion of the respondents (14.3%) achieved a score below or equal to 5. The patient-physician relationship scores ranged from 0 to 20, the mean score was 16 and the standard deviation (SD) was 4.3. The better the patient-physician relationship, the more information was finally delivered to the patient from the physician (Spearman's rank-order correlation coefficient was 0.4 and p<0.001). Delivered information index was significantly higher among participants who comprehended the right to informed consent, compared to participants who did not (p<0.001), and among participants who were given information regarding other possible therapeutic options (p = 0.001). 43% of the respondents answered that less than 10 minutes were spent on the consent process, 58.4% of patients stated that they had not been informed about other possible therapeutic choices and 28.6% did not really comprehend their legal rights to IC.

**Conclusions:**

Despite the inherent limitations and the small sample size that do not permit to draw any firm conclusions, results indicate that a successful IC process may be associated with specific elements such as the patient-physician relationship, the time spent by the physician to inform the patient, a participant's comprehension of the right to IC and the provision of information regarding other possible therapeutic options.

## Introduction

Certain aspects of obtaining and giving informed consent (IC) became an issue in biomedical ethics for the first time during a period stretching from the mid-17th to the early 19th century; IC was concerned with the same principles that prevail today [1]. Nowadays, informed consent has replaced the old paternalistic notion of “the doctor knows best”, with a more collaborative patient-physician relationship. Patients expect to be informed of the risk of surgical interventions[2]. Communication is a key component especially in the case the patient has to weigh the risks and benefits of a recommended treatment, and the overall quality of patient care[3]. On the other hand, it seems that even though patients welcome the collaborative spirit, they may not all be interested in taking complete charge of their medical decisions[4], some prefer the physician to be the primary decision maker[5] and a few are even willing to surrender utter control to their physician[6], [7].

The most important goal of informed consent is to effectively inform patients about the recommendations and reasoning process of the doctor and help the patient make the final decision about their healthcare[8]. This process involves the discussion of several elements including the nature of the proposed medical intervention, duration of hospital stay, alternative therapeutic options, risks, benefits, inconveniences, and uncertainties related to each alternative. The doctor assesses the patients' level of comprehension and provides the information in a way and to an extent that satisfies the individual's needs and ensures that all questions have been answered. Finally, it should be clear that patients may change their mind at any point[8].

Information, regarding the procedure for which consent is asked, may be given orally, written, or both. Either method has certain advantages and drawbacks. Documents often present complex information that is hard to be understood by patients. On the other hand, verbal information is rather difficult to retain[7]. It should be mentioned that ethicists consider that a signed written form is not equivalent to IC itself. They believe that IC is a dialogue between doctor and patient. A written form promotes the dialogue process, and helps to ensure that the patient has talked with the doctor and agrees to proceed. The written form should not replace the personal contact and informed consent should not only be written.

In a truly successful IC, patients fully comprehend the procedure, their rights and responsibilities [9]. However, the amount and type of information that should be given to patients is questioned and many believe that too much information increases pre-surgery anxiety' [10]. For low-risk medical procedures physicians may not inform their patients in detail, however, consent should be a requisite' [11]. Finally, patients with poor literacy should be identified and the information provided should have adequate continuum, readability and comprehensibility[11].

The degree of physicians' control over the process of decision-making is controversial. Physicians should preferably act as navigators for their patients' decision-making by providing a reasonable amount of information that will help the patient comprehend the ramifications of choice. They should not make decisions for the patient, even if he or she wishes so. The consequences of a patient's choice cannot be shared, and medical decisions should not be shared with the doctor either. Perhaps, shared medical decision makes choices easier for the patient. However, this is not the goal of informed consent. Patients have to understand all the risks and uncertainties of their decision[5].

Greece was among the first European countries (1992) to enact legislation directly addressing the rights of mentally healthy patients to IC. However, partial measures were taken for the wide implementation of the legislation. Five years later, in 1997, patients' rights act were extended to impose the provision addressed by the law 2071/92[13]. Still, the Greek legislation has not set specific rules defining in detail the way IC should be communicated to patients. Thus, it is left to the judgment of physicians to choose the way to inform patients and acquire the latter's consent on performing medical procedures.

We sought to record and evaluate patients' views of the way surgeons communicate IC in the Greek health care setting. Furthermore, we aimed to record the information that patients really comprehended as well as their perception of the significance of IC. This is an exploratory pilot study.

## Methods

### Study Subjects

A pilot study was carried out from September 2007 to April 2008. The study sample was extracted from patients, operated by seven general surgeons (one working at a private hospital and six at a state hospital), and one ophthalmologist (working at a private hospital) in association with the Alfa Institute of Biomedical Sciences (AIBS) Athens, Greece. Surgeons asked certain patients to voluntarily participate in this survey. There was no specific protocol or methodology on the selection of the participants of this survey as this was a convenience sample. Written informed consent was taken by the participants and the study was approved by the Ethics Comittee of AIBS in collaboration with Hellenic American University. According to Greek institutional policies, patients signed a legal form that certifies that they have been informed on the nature, risks, and benefits of the surgery and that they consent to all required medical procedures including anaesthesia.

### Study Questionnaire

Self-report measures are important tools for understanding the IC process[14]. We developed a questionnaire of several items (Appendix S1), organized in four parts: a) questions regarding general demographic information (gender, marital status, age, education level, profession, ethnicity and place of residence) (Appendix S1 - general information questions 1–8); Questions about the number of children, the place of residence and the profession of the respondents were not used in the analysis b) questions regarding the information delivered to the patient through the IC process (substantial elements of information that should be provided by the surgeon and recalled by the patient) (Appendix S1 – part I questions 1,2,4–11); c) questions regarding the perception of significance, the proper application and comprehension of the IC process (Appendix S1 – part II questions 1–9); d) questions regarding the patient-physician relationship (Appendix S1 – part III questions 1–9) and respondents' opinion on the questionnaire itself (Appendix S1 – part III question 10). The questionnaire was developed taking into consideration current literature regarding the goals and requirements for IC. There was only one version that was used in this pilot study.

A research assistant was available at all times to assist the participants to complete the questionnaire. The assistant answered questions regarding the comprehension of the questionnaire.

### Data Analysis

To better serve the purposes of this exploratory pilot study we constructed a composite delivered information index. One point was given for every “positive” answer (answer indicating that the certain element of information was successfully delivered to the patient) to the 10 questions presented in Table 1 (Appendix S1 part 1 questions 1–2, 4–11). The delivered information index ranged from 0–10. Furthermore, we constructed a respective patient-physician relationship index using a Likert type scale methodology. Responses to the five questions presented in Table 2 (Appendix S1 part III questions 2–6) were scored and added. The patient-physician relationship index ranged from 0–20. Categories of education were combined; participants were divided into 2 groups; the first had secondary education or higher, and the second group had elementary education or lower. The eight participating surgeons were grouped into those practicing in a private hospital (one general and one ophthalmic surgeon) and those practicing in a state hospital (six general surgeons).

**Table 1:** Responses to questions of the composite delivered information index*.

Questions	Yes Number (%)	No Number (%)
Answers		
**Are you aware of your problem and diagnosis?**	77 (100)	0
**Are you aware of why you are having this operation?**	77 (100)	0
**Were you informed about the duration of your hospital stay?**	58 (75.3)	19 (24.7)
**Did you feel that the inconveniences and potential risks of the operation were explained?**	57 (74)	20 (26)
**Were the risks explained in case you decided against the operation?**	59 (76.6)	18 (23.4)
**Were the potential benefits of the operation explained?**	68 (88.3)	9 (11.7)
**Were post-operative issues (such as complications) discussed?**	55 (71.4)	22 (28.6)
**Where you informed about the duration of your treatment?**	54 (70.1)	29.9 (29.9)
**Did you receive too much information?**	52 (32.5)	25 (67.5)
**Were you satisfied with the amount of the information you received?**	64 (83.1)	13 (16.9)

* Appendix S1 part 1 questions 1–2, 4–11.

**Table 2:** Responses to questions investigating the patient-physician relationship*.

Questions	Never Number (%)	Seldom Number (%)	Sometimes Number (%)	Often Number (%)	Always Number (%)
Answers					
**Do you trust your surgeon?**	0	1 (1.3)	2 (2.7)	13 (17.3)	59 (78.7)
**Do you feel comfortable with your surgeon?**	0	1 (1.4)	8 (11.4)	15 (21.4)	46 (65.7/4)
**Do you respect your surgeon's opinion?**	0	0	1 (1.4)	7 (9.7)	64 (88.9)
**Did you express your concerns about the operation to the surgeon?**	11 (16.2/0)	2 (2.9)	12 (17.6)	6 (8.8)	37 (54.4)
**Did you feel that the surgeon heard and understood you opinions and concerns?**	2 (3/0)	0	3 (4.5)	10 (15.3/3)	51 (77.3)

* Appendix S1 part III questions 2–6.

### Statistical Methods

Mann-Whitney U test was used to detect differences in the patient-physician relationship index and delivered information index (Table 3) when the sample was grouped according to gender, age, and education level (Appendix S1 general information questions 1, 3, and 5), comprehension of the right to IC, delivery of information regarding other therapeutic choice, perception of importance of IC (Appendix S1 part III questions 1,2 and 8), and surgery in state or private hospital (as derived by the grouping of the participating surgeons). Spearman's rank-order was used to measure the correlation between the indexes of delivered information and patient-physician relationship, as well as between delivered information index and age and finally between patient-physician relationship index and age. All reported P-values are based on two-sided tests and compared to a significance level of 5%. SPSS version 15.0 software (SPSS Inc. 2007, Chicago, Illinois, USA) was used for statistical calculations.

**Table 3:** Subgroup comparisons.

Grouping variable		Delivered information (mean±SD)	P	Patient-physician relationship (mean±SD)	P
Compared index					
**Gender:**	**Male**	8.6±1.8	**0.002**	15.9±4.5	0.69
	**Female**	7.5±1.8		16.3±4.1	
**Age:**	**<18**	9	0.9	19	0.6
	**18–25**	7.3±2		18.3±1.5	
	**26–35**	7.7±1.7		15.8±4.5	
	**36–45**	7.9±2.2		17.3±1	
	**46–55**	8±2.3		16.8±0.6	
	**56–65**	8.1±1.9		14.2±1.6	
	**>65**	7.9±2		16.2±0.6	
**Education level:**	**Lower**	7.6±2.1	0.38	15.4±5.2	0.9
	**Higher**	8.1±1.9		16.2±4.2	
**Comprehension of the right to IC:**	**Yes**	8.5±1.6	**<0.001**	16.4±4.6	**0.012**
	**No**	6±1.5		14.5±2.8	
**Other therapeutic options given:**	**Yes**	8.9±1	**0.001**	17±4.3	0.053
	**No**	7.2±2		15.6±4	
**Perception of IC:**	**Important**	8.2±1.8	0.62	17±3.3	0.26
	**Not important**	8±1.9		15.5±5	
**Operated in a:**	**State hospital**	7±2	**<0.001**	17±4.5	**<0.001**
	**Private hospital**	8.8±1.2		14.8±3.7	

IC: informed consent, SD: standard deviation, all p values<0.05 are presented in bold fonts.

## Results

All patients approached agreed to complete the questionnaire. In total, 77 patients (42 males) operated by eight different surgeons, volunteered to respond to the questionnaire. Forty-three respondents (56%) were from 18 to 55 years old, only one was below 18, and the rest 33 (43%) were older than 55 years. Regarding the marital status, 22%, 57%, 10%, and 8% of the respondents were single, married, divorced and widowed, respectively. Nine percent (9%) of the respondents were of lower educational level (elementary school) while 91% of the respondents had at least secondary level education. Specifically, only 2.6% of the respondents did not graduate from the primary school, 40% were university graduates, 12% were technical school graduates. Ninety-six percent of participants were of Greek ethinicity, only three (4%) participants were non-Greeks.

The delivered information index score ranged from 3 to 10, the mean score was 8 and the standard deviation (SD) was 1.9. 14.3% of the respondents achieved a score between 3 and 5, 29.8% had a score between 6 and 8, 29.9% of the participants achieved a 9, and 26% of the respondents achieved the maximum score of 10. It should be stressed that all patients were aware of the underlying diagnosis and reasons of surgery (Appendix S1 part I questions 1 and 2). The rest of the questions of the composite delivered information index were affirmatively answered by a smaller proportion of the patients ranging from 32% to 88% (Table 1).

In Table 2, we present the responses to questions investigating the way patients perceive their surgeon's role. Almost 30% of the respondents stated that they trust, feel comfortable with, feel respectful towards and express their worries to the surgeon (they achieved a score of 20 in the patient-physician index). On the other hand, 16% of the respondents did not express their worries to the surgeon. The patient-physician relationship scores ranged from 0 to 20, the mean score was 16 and SD was 4.3. Furthermore, 27.3% of the respondents allocated a low score to their relationship with their surgeon (score equal or below 13).

In Table 3, we present in detail various subgroup comparisons for the patient-physician relationship and delivered information indexes. The delivered information index was significantly higher among males (p = 0.002). There was not any significant difference among participants of different age groups (Table 3). The mean delivered information index was higher among persons with secondary or higher education (8.1) compared to patients with primary school education or lower (7.6). However, the above difference was not statistically significant (p = 0.38). Furthermore, the delivered information index was significantly higher among participants that comprehended the right to informed consent, compared to participants that did not (p<0.001), and among participants who where given information regarding other possible therapeutic options (p = 0.001). Finally, patients operated in a private hospital achieved a higher delivered information index (mean score 8.8) compared to those operated in a state hospital (mean score 7) (p<0.001).

In Table 4, we present in detail answers to Part II of the questionnaire. Part II of the questionnaire investigated the perception of significance, the proper application and comprehension of the IC process; 38% of the respondents answered that less than 10 minutes were spent on the consent process, only 40.3% of patients stated that they had been informed about other possible therapeutic choices, and 19.5% did not really comprehend their legal rights to IC.

**Table 4:** Responses to questions regarding the perception of significance, the proper application and comprehension of the IC process.

Questions	Answers Number* (%)			
**Did you comprehend your rights concerning the informed consent?**	Yes 57 (74.7)	No 8 (10.4)	Not sure 7 (9.1)	N/A 4 (5.2)
**Where you informed about possible other therapeutic choices?**	Yes 31 (40.3)	No 21 (27.3)	Not sure 2 (2.6)	N/A 18 (23.4)
**What was the average time spent on this consent procedure with the surgeon/medical staff?**	Less than 5 minutes 15 (19.5)	5–10 minutes 14 (18.2)	More than 10 minutes 40 (51.9)	
**Did you understand all the parts of the consent form?**	Yes 61 (79.2)	No 7 (9.1)		
**Do you think you can change your mind once you gave your consent?**	Yes 25 (32.5)	No 31 (40.3)	Not sure 7 (7.8)	N/A 9 (11.7)
**How important do you think is the informed consent procedure?**	Very important 40 (51.9)	Important 29 (37.7)	Moderately important 2 (2.6)	Not Important 1 (1.3)

IC: informed consent, *out of 77 participants, N/A: not applicable.

The higher the patient-physician relationship index the higher the mean delivered information index (Spearman's rank-order correlation coefficient was 0.38 and p = 0.001). Finally, the patient-physician index was significantly correlated with the time spent on the IC process (Spearman's rank-order correlation coefficient 0.47 p<0.001).

## Discussion

One of the main findings of this exploratory pilot study is that information that should be delivered through the IC process did not reach patients in all cases (14.3% of the respondents achieved a delivered information index between 3 and 5 and 29.8% had a score between 6 and 8). Thus, the main goal, to get informed about the recommendation and reasoning process of the doctor, was not fully achieved. In most cases patients partially comprehended substantial information regarding the benefits, risks and inconveniences of the suggested treatment. This finding is in accordance with other investigators suggesting that, even though the health care provider has an ethical and legal responsibility to ensure comprehension of IC, it is unclear whether the means of communicating medical information to the patients are effective[15], [16].

Furthermore, this study showed that patients expressing higher degrees of satisfaction with their surgeon also reported higher levels of satisfaction with the IC process. One should be cautious with the interpreting these associations because these two variables may be merely confounded and both may simply be expressing satisfaction. We believe that a patient-physician relationship built on respect, open and honest communication, trust, and compassion promotes a more effective IC process. Patients who are trusting towards their physician, and physicians who work with patients to understand and involve them into the treatment plan are more likely to establish better communication. Through this process they choose the therapeutic scheme that best fits the patients' resources, needs, desires and ability to understand.

About half of inpatients awaiting investigation or treatment, or both, are unhappy with the amount of information received[17]. The relevant numbers for this study were considerably lower; 17% of the participants were not satisfied with the amount of information they received. However, respondents in our survey answered the questionnaire post-surgery and most probably had good outcomes. It should be noted that patients were not selected by independent observers and thus, this finding may be due to the selection of patients who were more satisfied and had better outcomes. Another interesting finding is that the time spent by the doctor to explain the procedure and inform the patient about the benefits, risks and inconveniences of the suggested treatment may directly be associated with the fulfillment of the IC goals.

The results of this study corroborate previous work indicating that many patients fail to recall major portions of information on consent. Patients' educational background is related to the level of attention they give to information provided by the physician and their ability to describe this information when asked. Despite the fact that most patients reported understanding all or most of the information, such communications are often too complex and difficult for many patients to grasp[8]. Perhaps simple consent for lower risk cases is indeed the most suitable for the shared decision-making process[11]. Participants with higher education grasped more information compared to patients with lower education. However, this study did not detect any statistical significance for the above correlation. It should be noted that the sample is not representative of the national educational level (almost 9% of the participants had elementary educational level compared to more than 40% of the general Greek population according to the 2001 population and housing census). The above finding may also reflect that doctors fail to provide material at appropriate educational levels. In addition, one cannot assume that a patient with a higher education level is necessarily “literate” regarding written forms or verbal information received[12].

Unfortunately, the Greek legislation has not set specific rules to define the minimum requirements for IC though general bioethical rules and laws do exist[15]. Thus, it comes without surprise that the mean delivered information index of patients operated by different surgeons ranged significantly. Surgeons' ability to communicate, and their subjective opinion on what patients are entitled to know may have defined their ability to inform their patients effectively. Of interest, surgeons who work in the private setting may deliver more information to their patients. However, the sample size is not large enough to draw any firm conclusions. There is a minimum on what information ought to be included in an IC, such as: nature of the procedure, including whether it is diagnostic or therapeutic, any risks involved, especially those that are severe and likely to occur, benefits of the procedure, and alternatives to the procedure, along with their risks and benefits[18]–[22]. The final goal is to fully engage the patients in their own health care decisions[23].

The most important consideration in the interpretation of the findings of our study relates to the small sample size. This is a pilot study and the preliminary results should be considered with caution. The study did not detect a significant correlation between the education level and the amount of the delivered information. This may be attributed to the fact that sampling was based on a volunteering process and illiterate persons may have been discouraged to participate given that the study included a questionnaire and not a personal interview by a researcher. The sample is not representative of the national attainment distribution. Furthermore, surgeons who participated in this study were not randomly selected and were aware of the purposes of this study. This may have introduced a certain degree of bias. Also, this is the first use of the constructed measures and they have not been validated. It should be acknowledged that the questionnaire was not anonymous and was answered at the hospital which may have influenced patients' answers. Of importance, in a univariate analysis the main limitation is that it does not permit to control for confounding factors to avoid a type 1 error. Thus, any inference made about cause and effect should be considered with cautiousness. Recall bias (this survey was conducted during the time after the patient was informed and they consented to the surgery), the effect of timing, the effect of the anesthesia, and the outcome of the surgery may have also been limiting factors. Finally, it should be noted that there was no specific protocol or methodology on the selection of the participants of this survey. Surgeons had different selection methods that may have been biased and may have selected participants based on their own perception of the level of literacy of the patient.

This pilot study contributes in a rather neglected area of research. To the best of our knowledge, this is the first study that investigates the implementation and implications of informed consent among surgical patients in Greece. Despite the inherent limitations and the small sample size that do not permit to draw any firm conclusions, results indicate that a successful IC process may be associated with certain elements such as the patient-physician relationship, the readability of the forms, the time spent by the physician to inform the patient, participant's comprehension of the right to IC and the provision of information regarding other possible therapeutic options. Finally, the fact that information that should be delivered through the IC process did not reach patients in all cases, stresses the need to implement rules that will better define the minimum requirements for IC. More research is needed on this topic in Greece and in other countries of similar socio-economic or political characteristics.

## Supporting Information

Appendix S1 (0.11 MB DOC)
