# Supplementary material for: Patients' Perception of Quality of Pre-Operative Informed Consent in Athens, Greece: A Pilot Study
Source: PLoS One. 2009 Nov 26;4(11):e8073. doi: 10.1371/journal.pone.0008073 (PMC2777312; doi:10.1371/journal.pone.0008073)
Supplement: Appendix S1 — (0.11 MB DOC) [file pone.0008073.s001.doc]

**APPENDIX – Survey tool**

**General information:**

1. **Gender:** 🞎 Male 🞎 Female
2. **Marital status:** 🞎 Single 🞎 Married 🞎 Divorced 🞎 Widow(er)
3. **Age:**  <18 🞎 18-25 🞎 26-35 🞎 36-45 🞎

46-55 🞎 56-65 🞎 > 65 🞎

1. **Children:** 🞎 No 🞎 Yes 🡪 how many?______
2. **Education level:** (Please check in accordance with last class you have completed)
   - Some grades of elementary school
   - Elementary School
   - Secondary School (high school/lyceum)
   - Undergraduate degree
   - Graduate degree
   - Technical School
   - Other:
3. **Profession:** _____________________________________
4. **City of permanent residence:** _____________________________________
5. **Ethnicity:** 🞎 Greek 🞎 Other:_______________________

**Part I**

|  |  | **Yes** | **No** | **Not sure** | **Not applicable** |
| --- | --- | --- | --- | --- | --- |
| 1. | Are you aware of your problem and diagnosis? |  |  |  |  |
| 2. | Are you aware of why you are having this operation? |  |  |  |  |
| 3. | **Who** informed you about the specific surgical procedure? (e.g. physician, nurse, etc) |  | | | |
| 4. | Where you informed about the duration of you **hospital stay**? |  |  |  |  |
| 5. | Did you feel that the **inconveniences and potential risks** of the operation were explained? |  |  |  |  |
| 6. | Were the risks explained in case you decided **against** the operation? |  |  |  |  |
| 7. | Were the potential **benefits** of the operation explained? |  |  |  |  |
| 8. | Were post-operative issues (such as complications) discussed? |  |  |  |  |
| 9. | Where you informed about the duration of your **treatment**? |  |  |  |  |
| 10. | Did you receive **too much** information? |  |  |  |  |
| 11. | Were you satisfied with the amount of the information you received? |  |  |  |  |

**Part ΙΙ**

|  | |  | | | **Yes** | | **No** | | **Not sure** | | | **Not applicable** |
| --- | --- | --- | --- | --- | --- | --- | --- | --- | --- | --- | --- | --- |
| 1. | | Did you comprehend your rights concerning the informed consent? | | |  | |  | |  | | |  |
| 2. | | Where you informed about possible other therapeutic choices? | | |  | |  | |  | | |  |
|  | |  | | |  | |  | |  | | |  |
|  | | | | | **Less than five minutes** | | | | **5-10 minutes** | | | **More than ten minutes** |
| 3. | | What was the **average time** spent on this consent procedure with the surgeon/medical staff? | | |  | | | |  | | |  |
|  | |  | | |  |  | | |  | | |  |
| 4. | | In your own words, what **do you understand** informed consent to be? | | | | | | | | | | |
|  | |  |  | | |  | |  | | |  | |
| 5. | | Did you understand all the parts of the consent form? | 🞎 **Yes** | | | 🞎 **No**  🡮 if “no” what do you think you did not understand? | | | | | | |
|  | | |  | | |  | | | | | | |
|  |  | | **Yes** | | | **No** | | **Not Sure** | | | **Not Applicable** | |
| 6. | Do you think you can change your mind once you gave your consent? | |  | | |  | |  | | |  | |
|  |  | |  | | |  | |  | | |  | |
| 7. | Did you ask any questions concerning the operation? | | 🞎 **Yes** | | | 🞎 **No**  Check the reason(s) below 🡮 | | | | | | |
|  | | |  | | | 🞎 It was all very clear to me | | | | | | |
|  |  |  |  |  |  | 🞎 No time | | | | | | |
|  |  |  |  |  |  | 🞎 Felt pressured by medical staff | | | | | | |
|  |  |  |  |  |  | 🞎 Other | | | | | | |
|  | |  |  | | |  | | | |  | |  |
|  | | | **Very important** | | | **Important** | | | | **Moderately important** | | **Not important** |
| 8. | How **important** do you think is the informed consent procedure? | |  | | |  | | | |  | |  |
|  |  | |  | | |  | | | |  | |  |
| 9. | I believe that the consent procedure is: (check ONE) | | | | |  | | | | | | |
|  | 🞎 **Important** (Circle BEST answer)🡾 | | | | | 🞎 **Not Important** (Circle BEST answer)🡾 | | | | | | |
|  | 1. Because it provides me with   information that helps me make-up  my mind better | | | | | A.) Because I’ll do as the surgeon says, independently of the informed consent | | | | | | |
|  | B.) Because it is a legal matter (it  provides legal protection to the  physicians) | | | | | 1. Because I have already decided I am   going to have the operation | | | | | | |
|  | C.) Other: | | | | | C.) Other: | | | | | | |
|  |  | | |  | |  | |  | | |  | |

**Part IΙI**

|  |  |  |  | |  | | | | | |
| --- | --- | --- | --- | --- | --- | --- | --- | --- | --- | --- |
| 1. | How long have you known your surgeon? 🡺 | Years ______ Months ______ Days ____ | | | | | | | | |
|  |  | **Always** | | **Often** | | **Sometimes** | **Seldom** | | **Never** | |
| 2. | Do you trust your Doctor? |  | |  | |  |  | |  | |
| 3. | Do you feel uncomfortable with your surgeon? |  | |  | |  |  | |  | |
| 4. | Do you respect your surgeon’s opinion? |  | |  | |  |  | |  | |
| 5. | Did you express your concerns about the operation to the surgeon? |  | |  | |  |  | |  | |
| 6. | Did you feel that the surgeon heard and understood you opinions and concerns? |  | |  | |  |  | |  | |
|  |  | **Yes** | | **No** | | **Not sure** | **Not applicable** | | | |
| 7. | Did you feel any pressure to consent to the proposed trial? |  | |  | |  |  | | | |
| 8. | If yes, from whom did you feel pressured? |  | | | | | | | | |
|  |  |  | |  | |  |  | | |  |
| 9. | Please indicate the importance of your relationship with your surgeon in you deciding to participate in this study (circle one) | 0 1 2 3 4 5  **Not at all Very much** | | | | | | | | |
|  |  |  |  | |  | | |  | |  |

**Additional Comments:**

__________________________________________________________________________________________________________

**OPTIONAL SECTION**

|  |  |  |  |  |  |  |
| --- | --- | --- | --- | --- | --- | --- |
| 10. | How easy did you find the above questionnaire? **(circle one*🡭)*** | “**Not easy**” 0 1 2 3 4 5 **“Very easy”** | | | | |

**Follow up information**

*We would like to follow-up with you for any future research related to this topic. With your permission, please provide us with your contact information and preferred times of contact.*

Name: __________________________________

Telephone(s): ______________________________

Address: _________________________________

*Best times to contact you?* 🞎 Anytime 🞎 Mornings 🞎 Afternoons 🞎 Evenings

***Please return the completed questionnaire to the staff or assistant when you are done. Thank you.***
